# Association between postoperative pathological results and non-sentinel nodal metastasis in breast cancer patients with sentinel lymph node-positive breast cancer

**DOI:** 10.1186/s12957-024-03306-8

**Published:** 2024-01-24

**Authors:** Lingguang Dong, Suosu Wei, Zhen Huang, Fei Liu, Yujie Xie, Jing Wei, Chongde Mo, Shengpeng Qin, Quanqing Zou, Jianrong Yang

**Affiliations:** 1 Department of Breast and Thyroid Surgery, Guangxi Academy of Medical Sciences, People’s Hospital of Guangxi Zhuang Autonomous Region, Nanning, Guangxi China; 2 Department of Scientific Cooperation of Guangxi Academy of Medical Sciences, People’s Hospital of Guangxi Zhuang Autonomous Region, Nanning, Guangxi China; 3 Scientific Research and Experimental Center, The People’s Hospital of Guangxi Zhuang Autonomous Region, Guangxi Academy of Medical Sciences, Nanning, Guangxi China

**Keywords:** Breast cancer, Sentinel lymph node, Non-sentinel lymph node, Metastasis

## Abstract

**Objective:**

For patients with 1–2 positive sentinel lymph nodes (SLN) identified by biopsy, the necessity of axillary lymph node dissection (ALND) remains a matter of debate. The primary aim of this study was to investigate the association between postoperative pathological factors and non-sentinel lymph node (NSLN) metastases in Chinese patients diagnosed with sentinel node-positive breast cancer.

**Methods:**

This research involved a total of 280 individuals with SLN-positive breast cancer. The relationship between postoperative pathological variables and non-sentinel lymph node metastases was scrutinized using univariate, multivariate, and stratified analysis.

**Results:**

Among the 280 patients with a complete count of SLN positives, 126 (45.0%) exhibited NSLN metastasis. Within this group, 45 cases (35.71%) had 1 SLN positive, while 81 cases (64.29%) demonstrated more than 1 SLN positive. Multivariate logistic regression analysis revealed that HER2 expression status (OR 2.25, 95% CI 1.10–4.60, *P* = 0.0269), LVI (OR 6.08, 95% CI 3.31–11.14, *P* < 0.0001), and the number of positive SLNs (OR 4.17, 95% CI 2.35–7.42, *P* < 0.0001) were positively correlated with NSLNM.

**Conclusion:**

In our investigation, the risk variables for NSLN metastasis included LVI, HER2 expression, and the quantity of positive sentinel lymph nodes. However, further validation is imperative, including this institution, distinct institutions, and diverse patient populations.

## Introduction

As of 2020, female breast cancer has surpassed lung cancer as the most prevalent form of cancer worldwide. In most nations, breast cancer stands as the primary cause of mortality and morbidity among women. Globally, 2,261,419 new cases and 684,996 deaths from female breast cancer were documented in 2020. Females constituted 24.5% and 15.5% of new cancer cases and deaths, respectively [1]. Breast cancer cells within the sentinel lymph node (SLN) can affect both the SLN and distant organs, similar to other cancers. Neglecting or mishandling breast cancer cells can lead to rapid dissemination [2].

The prevailing treatment approach for breast cancer predominantly involves a blend of surgical interventions. Sentinel lymph node biopsy (SLNB), which has progressed rapidly in recent years, is now the primary method, gradually supplanting axillary lymph node dissection (ALND) for evaluating axillary lymph node (ALN) metastases [3].

SLNB has augmented physicians’ ability to determine disease staging, predict prognosis, and offer optimal treatment strategies, aiming to enhance patients’ quality of life and positive outcomes [4, 5]. Notably, overall survival and relapse-free survival remain unaffected by complete axillary lymph node excision post-SLNB, prompting a tendency toward conservative axillary management and treatment. SLN biopsy demonstrates comparable success to ALND in terms of staging, locoregional control, and survival without lymphedema and swelling [6]. In instances of a positive SLNB test during surgery, it is customary to prescribe planned ALN excision either concurrently with SLNB surgery or in subsequent therapy to improve prognosis [7]. Controversy surrounds the use of ALND in patients with one or two positive SLNs, as skipping the operation appears to have no impact on clinical outcomes [8]. Moreover, scant investigations have explored the link between 1–2 positive SLNs and ALN metastases in Chinese breast cancer patients. Hence, identifying predictor variables for ALN status in women with breast cancer is imperative, particularly for those with 1–2 positive SLNs who may or may not require immediate major surgery. Risk factors such as age, body mass index (BMI), tumor size, progesterone receptor status (PR status), estrogen receptor status (ER status), human epidermal growth factor receptor-2 status (Her-2 status), and other clinical indicators have been identified in numerous previous studies as linked to ALN metastasis in breast carcinoma [9–11]. Despite this, ethnic/racial disparities in ALN metastasis incidence appear to have an inconsistent impact on these factors [10]. Therefore, comprehending the risk variables associated with ALN status, especially in Chinese patients with breast carcinoma, is essential for researchers.

Numerous studies have proposed that the presence of a positive SLN constitutes a predisposing factor for metastasis to non-sentinel lymph nodes (NSLNs) [12]. Upon identification of a positive SLN, it is conventionally imperative to undertake ALND to stop tumor recurrence and metastasis, thereby improving the overall prognosis of the afflicted individual. However, certain investigations proposed that it might be judicious to avoid ALND in cases where one or two positive SLNs are detected [13]. Nevertheless, the veracity of these findings remains subject to ongoing debate. To assure tumor safety, ALND remains the established procedural norm in the western region of China when positive SLNs are ascertained. To comprehensively elucidate the risk factors related to postoperative pathology and the occurrence of metastasis in NSLNs among this cohort, and to inform clinical decisions on whether ALND may be omitted, this study categorically stratified cases based on the quantity of positive SLNs. This investigation involved 280 Chinese patients afflicted with breast cancer characterized by positive SLNs. Univariate and multivariate logistic regression analyses were conducted to ascertain the correlation between the number of positive SLNs and the occurrence of non-sentinel lymph node metastasis (NSLNM) in women with breast cancer. The objective was to identify factors that independently correlate with NSLNM. The findings herein may serve to delineate high-risk indicators for NSLNM.

## Materials and methods

### Participants

**Fig. 1:**
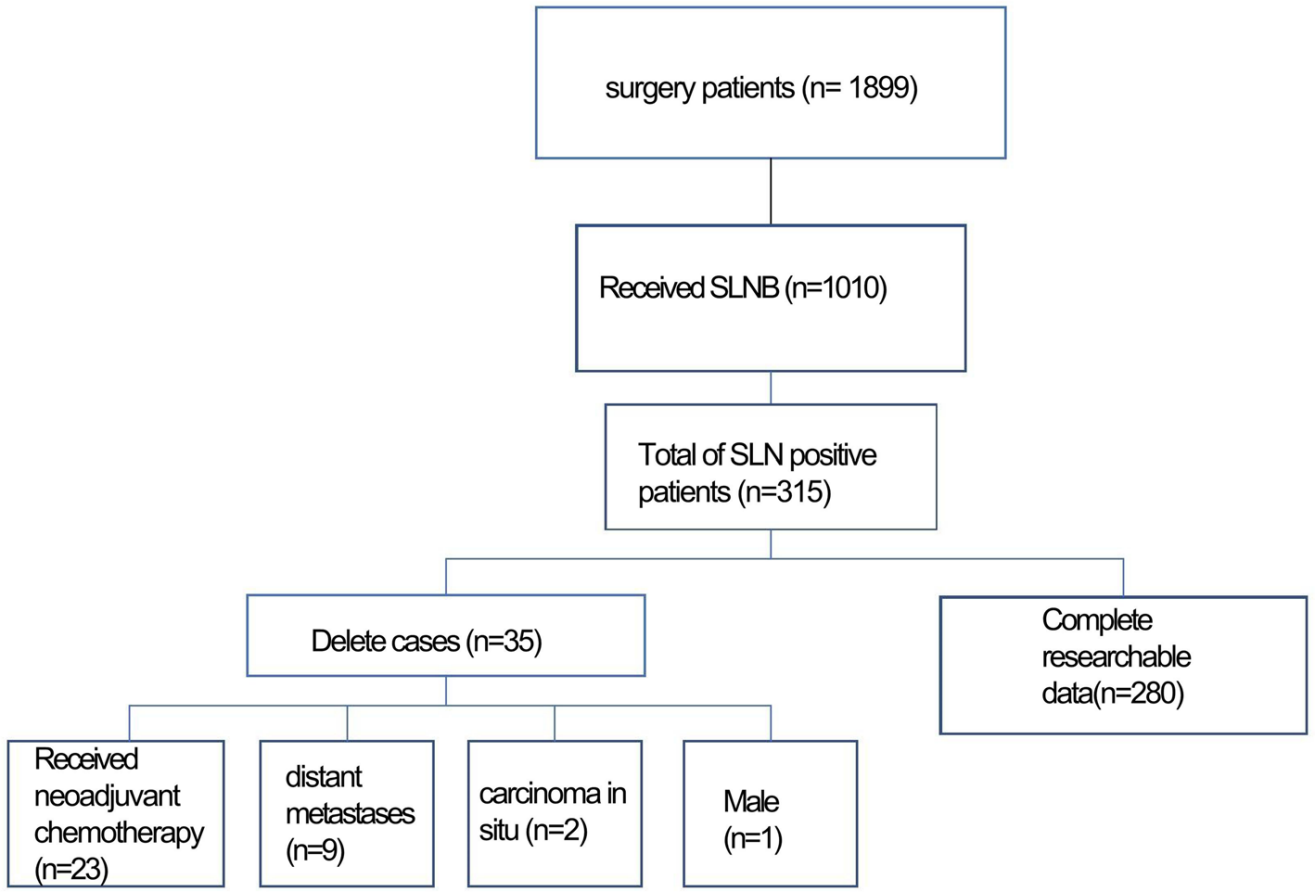
The flow chart of the study

This study included patients who underwent breast cancer surgery at the Breast Department of the Guangxi Zhuang Autonomous Region People’s Hospital between March 7, 2013, and July 4, 2022, provided they presented with SLN-positive breast cancer. Exclusions were made for cases involving neoadjuvant chemotherapy, carcinoma in situ, males, and instances of distant metastases. Notably, none of the enrolled patients underwent systemic treatment before the surgical intervention. A total of 280 patients, who underwent SLNB accompanied by ALND, were thus incorporated into our investigation. Histological verification of the diagnosis was achieved through preoperative needle core biopsy or intraoperative frozen section. Preoperative biopsies of potentially metastatic lymph nodes were conducted based on imaging and SLNB findings. Blue dye injection marked the SLN before the surgical incision. Patient treatment involved either complete mastectomy or breast-conserving surgery. Sentinel lymph nodes labeled with methylene blue underwent frozen section analysis. In cases where significant or micrometastases were identified in the SLN through frozen section analysis, ALND was undertaken. Both preoperative puncture pathology and intraoperative freezing pathology results harbored the potential for false negatives, with the conclusive diagnosis relying on routine postoperative pathology results. However, in the event of false negatives, ALND was performed during a subsequent procedure. In instances of SLNB failure, ALND was deemed unavoidable. If SLN pathology revealed isolated tumor cells (ITCs), ALND was omitted. The study’s procedural pathway is depicted in the flow chart presented below (Fig. 1).

### Data collection

Requisite patient information, including age (< 50 or ≥ 50 years old), BMI, menopausal status, pathological tumor size (< 2 or ≥ 2 cm), histological grade (middle/low, high, unknown), Lymphovascular infiltration (LVI; yes or no), number of SLN positives, PR status (negative or positive), ER status (negative or positive), Ki-67 receptor status (< 14% is negative, ≥ 14% is positive, unknown), and HER2 status (negative or positive, unknown), may be acquired through computer systems, outpatient systems, and health data platforms. Subsequent to data acquisition, a retrospective analysis was conducted.

The study further categorized the quantity of positive SLNs to examine the association between these nodes and NSLNM. Concurrently, an investigation was undertaken to scrutinize the correlation between postoperative pathological variables and NSLNM in patients afflicted with SLN-positive breast cancer.

## Methods

Upon initiation of general anesthesia, successive incisions were made through the skin and subcutaneous tissue. Intraoperatively, methylene blue was employed to discern SLNs, with any lymph node displaying a blue hue considered an SLN. Following SLN identification, frozen sections were prepared. The preserved stump tissues in paraffin-embedded slices underwent hematoxylin and eosin (H&E) staining. Affirmative results prompted ALND. Evaluation of all axillary lymph nodes (both SLNs and NSLNs) was conducted using H&E staining and paraffin histopathology. The decision for adjuvant ALND was contingent upon the findings from either intraoperative frozen sections or postoperative paraffin histopathology. The study was founded on a cohort of approximately 280 patients with comprehensive data, each having at least one positive SLN.

The determination of breast cancer tumor size in this study relied upon the maximal diameter of the lesion, adhering to the diagnostic criteria set forth by the American Joint Committee on Cancer (AJCC) and the World Health Organization (WHO). Conforming to the AJCC's 8th edition standards, the designations are as follows: Tx denotes that the primary tumor is not evaluable, Tis indicates carcinoma in situ and Paget's disease without a lump, T0 signifies no evidence of a primary tumor. T1 designates a tumor with a diameter ≤ 2 cm, T2 indicates a maximum tumor diameter > 2 cm and ≤ 5 cm, T3 signifies a maximum tumor diameter > 5 cm, and T4 is defined as a tumor of any size that directly invades the chest wall and/or skin; however, dermal invasion alone does not qualify as T4. The WHO diagnostic criteria categorize breast cancer into three grades: I, II, and III. Grade I denotes low-grade malignancy and high tissue differentiation, grade II signifies moderately malignant and moderately differentiated, while grade III represents highly malignant and poorly differentiated. Pathological parameters were evaluated in accordance with established guidelines. The determination of ER, PR, and HER2 receptor presence was conducted on resected primary tumors or core biopsy samples. Positivity for ER or PR was affirmed when PR and ER were > 1%. HER2 expression was assessed via immunohistochemistry (IHC). A HER2 status of 3 + indicated positive expression, while a status of 1 + or 0 signified negative expression. In cases where HER2 status could not be determined through immunohistochemistry, gene amplification assays were employed using fluorescence in situ hybridization (FISH). A HER2 status of 2 + with FISH amplification indicated positive expression, whereas no FISH amplification indicated negative expression. Uncertainty was designated if the patient declined the FISH test and HER2 expression was 2 + . Immunohistochemistry was utilized to quantify Ki-67 protein expression, with outcomes presented as the percentage of cancer cells exhibiting antibody staining. Missing Ki-67 data is reported as unknown. LVI was evaluated using methylene blue staining.

### Statistical analysis

In accordance with the prevailing circumstances, data were presented either as mean ± SD or median (quartiles). Categorical data were expressed using percentages and frequencies. To facilitate comparisons between the two groups, Mann–Whitney* U* tests were employed for continuous variables, while Pearson's *χ*2 test was applied for categorical variables. The association between various clinicopathological variables and NSLN metastases was assessed through multivariable logistic regression models. A multicollinearity test was executed to examine the correlation between independent variables, utilizing collinearity statistics (variance inflation factors > 5 were deemed suggestive of multicollinearity). Unadjusted and adjusted odds ratios (OR) with 95% confidence intervals (CI) were calculated. Stratified analyses were conducted based on age, ER, HER2, histological stage, Ki-67, LVI, PR, menopausal status, and tumor size. Data analysis was performed using the statistical software program SPSS 18 (IBM Corp, Armonk, NY, USA). Significance was attributed to statistics with a *p* value < 0.05.

## Results

### Baseline features of the people in the study

Following stringent inclusion criteria, as delineated in Fig. 1, a total of 280 patients meeting the specified criteria were included in the study. All participants were female. Table 1 presents the clinical and pathological features of these patients at baseline. At the time of diagnosis, the mean patient age was 50, with an average BMI of 23.11 kg/m^2^. Of the total, 163 (58.21%) were premenopausal, and 117 (41.79%) were postmenopausal. On average, patients had one SLN. Table 1 also notes that in cases where HER2 status was unknown, the patient’s HER2 expression was recorded as 2 + , and she declined to undergo a fluorescence in situ hybridization (FISH) test. Additionally, instances labeled as unknown in histological grade and Ki-67 denoted missing data. The table further delineates various postoperative pathological characteristics.

**Table 1:** Baseline characteristics of subjects(*n* = 280)

Characteristic	Patients
Age	50.00 (44.00–58.00)
BMI	23.11 (21.50–25.57)
Number SLNM	1.00 (1.00–2.00)
Histological stage	
Midddle/low	231(82.50%)
High	25(8.93%)
Unknown	24(8.57%)
Tumor size (cm)	
≤ 2	93 (33.21%)
> 2	187 (66.79%)
Ki-67	
Negative	203 (72.50%)
Positive	75 (26.79%)
Unknown	2 (0.71%)
HER2	
Negative	215 (76.79%)
Positive	63 (22.50%)
Unknown	2 (0.71%)
ER	
Negative	47 (16.79%)
Positive	233 (83.21%)
PR	
Negative	77 (27.50%)
Positive	203 (72.50%)
LVI	
Negative	136 (48.57%)
Positive	144 (51.43%)
menopause	
No	163 (58.21%)
Yes	117 (41.79%)

*BMI* body mass index, *Number SLNM* the number of sentinel lymph node metastases, *HER2* human epidermal growth factor Receptor 2, *ER* estrogen receptor, *PR* progesterone receptor, *LVI* lymphovascular invasion

### Relationship between the postoperative pathological results in SLN-positive cases and NSLN metastasis

Tables 2 and 3 elucidate the association between postoperative pathological results in SLN-positive cases and NSLN metastasis. Among the 280 patients, 126 manifested NSLN metastasis, while 154 exhibited no metastasis after ALND. The outcomes of single-factor analysis are delineated in Table 2, wherein tumor size, HER2 expression status, LVI, and the number of positive SLNs emerged as reliable indicators of NSLN metastasis, demonstrating statistical significance (*p* < 0.05). Table 3 presents the results of univariate and multivariate analyses after eliminating unknown data. The final outcome of the single-factor analysis aligns with the conclusion drawn from the single-factor analysis (Table 2). Notably, a substantial correlation between NSLN metastasis and tumor size (OR 1.93, 95% CI 1.15–3.22, *P* = 0.0126), HER2 expression status (OR 2.05, 95% CI 1.16–3.63, *P* = 0.0134), LVI (OR 6.11, 95% CI 3.62–10.32, *P* < 0.0001), and the number of positive SLNs (OR 4.50, 95% CI 2.72–7.46, *P* < 0.0001) was established. Furthermore, in the multifactor analysis, tumor size exhibited no association with non-sentinel lymph node metastasis. Conversely, reliable indicators of NSLN metastasis included HER2 expression status (OR 2.25, 95% CI 1.10–4.60, *P* = 0.0269), LVI (OR 6.08, 95% CI 3.31–11.14, *P* < 0.0001), and the number of positive SLNs (OR 4.17, 95% CI 2.35–7.42, *P* < 0.0001). No statistically significant differences were identified for age, BMI, menopause, histological grade, Ki-67, ER, or PR (Table 2 and 3).

**Table 2:** Correlation between non-sentinel lymph node metastasis and clinicopathological features

Variables	No metastasis (*n* = 154)	Metastasis (*n* = 126)	*P*-value
Age			0.524
≤ 50	76 (49.35%)	67 (53.17%)	
> 50	78 (50.65%)	59 (46.83%)	
BMI			0.378
≤ 25	105 (68.18%)	92 (73.02%)	
> 25	49 (31.82%)	34 (26.98%)	
Tumor size (cm)			0.012 *
≤ 2	61 (39.61%)	32 (25.40%)	
> 2	93 (60.39%)	94 (74.60%)	
Histological stage			0.580
Midddle/low	124 (80.52%)	107 (84.92%)	
High	16 (10.39%)	9 (7.14%)	
Unknown	14 (9.09%)	10 (7.94%)	
Ki-67			0.243
Negative	115 (74.67%)	88 (69.84%)	
Positive	37 (24.03%)	38 (30.16%)	
Unknown	2 (1.30%)	0 (0.00%)	
HER2			0.044 *
Negative	127 (82.47%)	88 (69.84%)	
Positive	26 (16.88%)	37 (29.37%)	
Unknown	1 (0.65%)	1 (0.79%)	
ER			0.962
Negative	26 (16.88%)	21 (16.67%)	
Positive	128 (83.12%)	105 (83.33%)	
PR			0.861
Negative	43 (27.92%)	34 (26.98%)	
Positive	111 (72.08%)	92 (73.02%)	
LVI			< 0.001 *
Negative	104 (67.53%)	32 (25.40%)	
Positive	50 (32.47%)	94 (74.60%)	
Number SLNM			< 0.001 *
1	110 (71.43%)	45 (35.71%)	
> 1	44 (28.57%)	81 (64.29%)	
Menopause			0.874
No	89 (57.79%)	74 (58.73%)	
Yes	65 (42.21%)	52 (41.27%)	

*BMI* body mass index, *Number SLNM* the number of sentinel lymph node metastases, *HER2* human epidermal growth factor Receptor 2, *ER* estrogen receptor, *PR* progesterone receptor, *LVI* lymphovascular invasion

^*^Statistically significant (*p* < 0.05)

**Table 3:** Univariate and multivariate logistic regression analysis for non-SLN metastasis

Exposure	Univariable		Multivariable	
	OR(95%CI)	*P*	OR(95%CI)	*P*
Age				
≤ 50	1		1	
> 50	0.86 (0.54, 1.37)	0.5243	0.85 (0.36, 2.01)	0.7172
BMI				
≤ 25	1		1	
> 25	0.79 (0.47, 1.33)	0.3786	0.73 (0.38, 1.40)	0.345
Tumor size (cm)				
≤ 2	1		1	
> 2	1.93 (1.15, 3.22)	0.0126 *	1.22 (0.65, 2.27)	0.5308
Histological stage				
Negative	1		1	
Positive	0.65 (0.28, 1.54)	0.3275	1.21 (0.43, 3.39)	0.7196
Ki-67				
Negative	1		1	
Positive	1.34 (0.79, 2.28)	0.2774	1.05 (0.53, 2.11)	0.8822
HER2				
Negative	1		1	
Positive	2.05 (1.16, 3.63)	0.0134 *	2.25 (1.10, 4.60)	0.0269 *
ER				
Negative	1		1	
Positive	1.02 (0.54, 1.91)	0.9615	1.23 (0.40, 3.76)	0.7127
PR				
Negative	1		1	
Positive	1.05 (0.62, 1.78)	0.8612	1.29 (0.51, 3.26)	0.5919
LVI				
Negative	1		1	
Positive	6.11 (3.62, 10.32)	< 0.0001 *	6.08 (3.31, 11.14)	< 0.0001 *
Number SLNM				
1	1		1	
> 1	4.50 (2.72, 7.46)	< 0.0001 *	4.17 (2.35, 7.42)	< 0.0001 *
Menopause				
No	1		1	
Yes	0.96 (0.60, 1.55)	0.8742	1.69 (0.72, 4.00)	0.2296

*BMI* body mass index, *Number SLNM* the number of sentinel lymph node metastases, *HER2* human epidermal growth factor Receptor 2, *ER* estrogen receptor, *PR* progesterone receptor, *LVI* lymphovascular invasion

^*^Statistically significant (*p* < 0.05)

Table 4 presents the findings of the stratified analysis regarding the relationship between the number of positive SLNs and NSLN metastases. The observed link in the stratified analysis aligns with that determined through the multivariable logistic regression analysis.

**Table 4:** Stratified analysis of NSLN metastases

Variables	Number SNM(*n* = 1)		Number SNM(*n* > 1)		OR(95%CI)	
	total	metastasis	total	metastasis	Crude	Mutually adjusted
Age						
≤ 50	71	19	72	48	5.47 (2.67, 11.23)	4.13 (1.74, 9.82)
> 50	84	26	53	33	3.68 (1.79, 7.58)	4.28 (1.82, 10.11)
BMI						
≤ 25	109	33	88	59	4.69 (2.56, 8.57)	4.10 (2.03, 8.31)
> 25	46	12	37	22	4.16 (1.64, 10.53)	4.51 (1.50, 13.60)
Tumor size (cm)						
≤ 2	65	15	28	17	5.15 (1.99, 13.36)	5.37 (1.77, 16.31)
> 2	90	30	97	64	3.88 (2.11, 7.12)	3.88 (1.88, 8.02)
Histological stage						
Midddle/low	126	36	105	71	5.22 (2.97, 9.16)	4.86 (2.54, 9.30)
High	15	6	10	3	0.64 (0.12, 3.53)	inf
Ki-67						
Negative	113	29	90	59	5.51 (3.01, 10.11)	4.75 (2.37, 9.54)
Positive	41	16	34	22	2.86 (1.12, 7.35)	4.53 (1.27, 16.20)
HER2						
Negative	123	33	92	55	4.05 (2.28, 7.22)	3.36 (1.74, 6.51)
Positive	31	12	32	25	5.65 (1.87, 17.10)	11.68 (2.01, 67.82)
ER						
Negative	27	6	20	15	10.50 (2.70, 40.88)	26.73 (2.16, 331.09)
Positive	128	39	105	66	3.86 (2.24, 6.67)	3.41 (1.81, 6.42)
PR						
Negative	45	11	32	23	7.90 (2.83, 22.07)	7.37 (1.85, 29.38)
Positive	110	34	93	58	3.70 (2.07, 6.63)	3.32 (1.71, 6.46)
LVI						
Negative	89	14	47	18	3.33 (1.47, 7.55)	2.81 (1.17, 6.73)
Positive	66	31	78	63	4.74 (2.26, 9.96)	5.07 (2.18, 11.81)
menopause						
No	81	20	82	54	5.88 (2.98, 11.62)	5.39 (2.44, 11.87)
Yes	74	25	43	27	3.31 (1.51, 7.24)	3.60 (1.33, 9.71)

Each stratification adjusted for all the factors (Age, BMI, ER, HER2, Histological stage, Ki-67, PR, LVI, menopause and Tumor size) except the stratification factor itself

*BMI* body mass index, *Number SLNM* the number of sentinel lymph node metastases, *HER2* human epidermal growth factor Receptor 2, *ER* estrogen receptor, *PR* progesterone receptor, *LVI* lymphovascular invasion

## Discussion

The primary objective of our study was to assess the risk factors for NSLN metastases in patients with sentinel node positivity, particularly focusing on the association between the quantity of positive sentinel nodes and the occurrence of metastases in non-sentinel nodes. Our findings indicate that the likelihood of non-sentinel node metastasis increases with the number of positive sentinel nodes. Moreover, we identified a correlation between HER2 expression and LVI in patients with sentinel node positivity and NSLN metastases.

Breast cancer staging and treatment decisions are significantly influenced by the status of the axillary nodes. ALN biopsy is a standard component of the staging procedure for breast cancer, particularly in cases with clinically negative axillaries. However, 40–70% of individuals with SLN positivity do not exhibit axillary metastases [14, 15]. Consequently, these patients may forego axillary clearance [8, 16]. Despite ALND being the prevailing standard of treatment for breast cancers with SLN metastasis, recent studies suggest that ALND may be unnecessary when only 1–2 sentinel nodes are positive in SLN-positive breast carcinoma patients. The ACOSOG Z0011 study demonstrated equivalent overall survival and recurrence rates for patients undergoing SLNB without ALND, compared to those undergoing ALND when only two or fewer sentinel nodes were involved [8]. Similarly, the Dutch AMAROS trial, which assessed ALND and radiotherapy in T1–T2 stage SLN-positive patients, reported comparable axillary control outcomes between the two modalities [17]. Consequently, numerous studies have explored variables associated with SLN metastasis and primary tumor histological characteristics. These investigations have identified various pathological characteristics of the underlying tumor and SLN linked to NSLN metastasis. However, there remains limited consensus on the risk factors for NSLN metastases. A meta-analysis by Van La Parra et al. identified key characteristics that may be utilized to predict non-sentinel metastases, including lymphatic vascular invasion in the primary tumor, extracapsular invasion, 1 or no negative lymph nodes, more than 2 positive sentinel lymph nodes, and metastasis size exceeding 2 mm [18].

In consideration of the histological attributes of primary tumors and their metastases, the NSLN status has been correlated with various factors. Notable among these factors are the size of the main tumor, the grade of the primary tumor, the maximum size of SLN positivity in SLN, PR/ER status, the presence of LVI, and the HER2 status. These variables are frequently scrutinized as potential risk factors in diverse research studies, yielding disparate conclusions. While some studies identify them as risk factors, others assert the lack of association between these factors and NSLN metastasis.

Numerous research studies have substantiated the association between tumor size and the probability of NSLN metastasis [19, 20]. Ozmen et al. demonstrated that tumor sizes exceeding 2 cm were correlated with elevated risks of lymph node metastasis [21]. According to Rao et al., T1 tumors obviate the necessity for axillary dissection, as the incidence of axillary lymph node involvement for tumors < 2 cm was 14.28%, contrasting with 61.11% for tumors > 2 cm [22]. Orang et al. indicated that in tumors < 1 cm [23], the occurrence of lymph node involvement was 2.8%. These investigations collectively suggest that smaller tumor dimensions are less prone to lymph node engagement. Recently, Dingemans et al. [24] and Wang et al. [25] ascertained that one determinant influencing NSLN metastasis is the size of the primary tumor. Conversely, some research did not discern a correlation between the size of the primary tumor and NSLN metastasis [12]. Analogously, the multivariate analysis in this study failed to disclose a relationship between the size of the primary tumor and NSLN metastasis. This observation could be attributed to the simplicity of our categorization and the absence of further subdivisions based on tumor size.

Studies indicate that individuals below the age of 60 exhibit an increased likelihood of developing ALN metastases, while patients aged 60 and above manifest a diminished risk of ALN metastases [26]. Notably, a limited number of research endeavors have identified a correlation between age and a positive NSLN when employing multiple variables [27, 28]. Concurrently, certain studies have failed to establish an association between age and NSLN metastasis, a trend also seen in the present investigation.

Obesity has been correlated with the occurrence and prognosis of breast malignancy, as documented in research [29]. Nevertheless, a recent meta-analysis [30] revealed that obese women exhibited an elevated incidence of breast cancer-specific mortality. Specifically, obese women demonstrated a 1.7-fold higher risk of stage III/IV disease compared to normal-weight counterparts, as indicated in a prior large-scale population-based case-cohort study. This study further identified that obesity seemed to impact breast cancer survival, partly attributable to positive lymph node status, larger tumor sizes, and distant metastases [31]. Notably, in overweight and obese women with breast carcinoma, BMI was found to potentially influence the MRI diagnosis of ALN invasion and axillary surgery [32]. These findings suggest a potential relationship between BMI and breast carcinoma metastasis to axillary lymph nodes. Regrettably, no association between BMI and breast cancer was discerned in the current research.

Limited studies have unveiled an association between ALN metastasis and menopausal status and histological grade, as also reported in this study. Certain investigations have indicated that the degree of cancer cell differentiation aligns with NSLN involvement. It has been reported that tumors exhibiting good differentiation are less prone to NSLN metastases. Moreover, earlier research has proposed that the molecular subtypes of breast cancer patients might serve as predictors for the development of NSLN metastases. Nevertheless, diverse studies have not consistently converged on the specific subtypes more predisposed to NSLN metastases [33, 34].

This study proffers evidence supporting a correlation between LVI and participation in NSLN. Conversely, Yıldız et al. [35] and Dozin et al. [36] have reported that LVI does not function as a risk factor for NSLN metastasis. Contrary to these findings, our research suggests a relationship between LVI and the dissemination of NSLN. Specifically, axillary NSLN metastases are more probable in SLN-positive patients exhibiting LVI infiltration.

Studies have posited that positive sentinel lymph nodes independently correlate with tumor diameter, lymphatic vascular infiltration, estrogen receptor, and Ki67. Among these factors, tumor diameter is identified as affecting NSLN metastasis, while lymphatic vascular infiltration, estrogen receptor, and Ki67 exhibit no significant correlation with NSLN metastasis [37]. The incidence of axillary lymph node involvement is increased in individuals with positive ER/PR status, as indicated by other research studies [38]. Notably, our investigation did not reveal a correlation between ER or PR positivity and NSLN metastasis. Wang et al. [39] demonstrated a strong association between NSLN metastasis and HER2 expression. Consistently, the findings of this study underscore that HER2 overexpression substantially elevates the likelihood of NSLN metastasis.

Our findings align with certain studies indicating that the number of positive sentinel lymph nodes is associated with the metastasis of NSLNs [40]. Predictors of NSLN metastasis, found in various research studies, include parameters such as the main tumor volume, the quantity of SLN-positive and SLN-negative nodes, tumor grade, SLN identification technique, LVI, ER/PR status, and tumor multifocality. Furthermore, to assess the probability of non-sentinel lymph node metastasis in patients with positive sentinel lymph nodes, some studies have devised prediction models based on these variables [41, 42]. However, it is pertinent to note that, as of the present, no universally recognized predictive model has been implemented in clinical practice.

Prior investigations have elucidated disparities in breast cancer outcomes across various racial and ethnic groups [43]. Notably, research indicates that, in comparison to other ethnicities, African Americans exhibit an increased prevalence of triple-negative breast cancer and experience a less favorable prognosis [2]. Furthermore, studies suggest that Jamaican women are afflicted by invasive breast cancer at a younger age, with a greater likelihood of ALN involvement, thereby adversely affecting prognosis [44]. These discrepancies may be attributed, in part, to variations in surgical interventions for axillary lymph nodes among individuals of different ethnic backgrounds, leading to statistical incongruities. Specifically, studies report a lesser prevalence of SLNB among African Americans in contrast to Caucasians [45]. Additionally, the rate of ALND is observed to be lower in non-White individuals [46]. However, it is noteworthy that controlling for lymph node surgery does not mitigate racial/ethnic disparities in overall survival or disease-specific survival, as evidenced by other research [47]. Consequently, further investigations are imperative to comprehensively explore the intricate associations between race, NSLNs, and patient outcomes.

In recent years, considerable discourse has surrounded the influence of SLN on NSLN metastasis. The prevailing consensus in many research findings suggests that a positive SLN serves as an independent predictor of NSLN metastasis. Nevertheless, there are divergent studies that have failed to establish a clear relationship between positive SLN and NSLN metastasis [19]. Notably, the outcomes of this study align with the majority of investigations, indicating that SLN positivity constitutes a risk factor for NSLN metastasis. Additionally, there is a suggestion that the quantity of negative lymph nodes may potentially serve as a prognostic factor for NSLN metastases. In essence, the more negative SLNs identified, the less probable NSLN involvement becomes [48]. Dong, L. F. et al. [49] noted a significant connection between NSLN metastasis in mammary cancer patients and the presence of positive SLN/negative SLN, while the quantity of SLN/negative SLN was statistically insignificant. Regrettably, this study did not incorporate negative SLNs, precluding an assessment of their impact on NSLN metastasis. Consequently, future research endeavors should address this aspect to deepen our understanding of the relationship between negative SLNs and NSLN metastasis.

The main limitation of our study is its retrospective nature, resulting in instances of missing data. Furthermore, the utilization of a single tracer in SLN staining may have led to the exclusion of certain patients with positive SLNs. Despite these limitations, our analysis, in concordance with the predominant findings in existing investigations, has delineated a correlation between sentinel node positivity, LVI, HER2 expression, and NSLN metastasis. It is imperative to conduct further validations of factors unrelated to NSLN metastasis not only in our facility but also across diverse facilities and among varied patient populations. This ongoing scrutiny is essential to enhance the robustness and generalizability of our findings.

## Conclusion

The risk factors identified for NSLN metastasis in our LVI, HER2 expression, and the quantity of positive SLNs. The present research does not definitively establish whether individuals with more than one SLN metastasis can avoid ALND. Drawing from these results, it is prudent to initiate pertinent cohort studies to delve deeper into the impact of these risk factors on the long-term prognosis of patients. Additionally, the findings provide a basis for the development of relevant prediction models, aiming to discern which SLN-positive patients may potentially be considered for exemption from ALND. This avenue of inquiry holds promise for refining clinical decision-making and tailoring treatment strategies based on individualized risk assessments.

## Data Availability

The datasets used and/or analyzed during the current study are available from the corresponding author on reasonable request.

## Abbreviations

SLN: Sentinel lymph node
SLNB: Sentinel lymph node biopsy
ALND: Axillary lymph node dissection
ALN: Axillary lymph node
BMI: Body mass index
PR: Progesterone receptor
ER: Estrogen receptor
HER2: Human Epidermal growth factor Receptor 2
LVI: Lymphovascular Invasion
NSLN: non-sentinel lymph node
ITC: Isolated tumor cells
SLNM: The number of sentinel lymph node metastases
FISH: Fluorescence in situ hybridization
